# Heterologous Expression, Purification and Immunoreactivity of the Antigen 5 from *Polybia paulista* Wasp Venom

**DOI:** 10.3390/toxins9090259

**Published:** 2017-08-24

**Authors:** Murilo Luiz Bazon, Amilcar Perez-Riverol, José Roberto Aparecido dos Santos-Pinto, Luis Gustavo Romani Fernandes, Alexis Musacchio Lasa, Débora Laís Justo-Jacomini, Mario Sergio Palma, Ricardo de Lima Zollner, Márcia Regina Brochetto-Braga

**Affiliations:** 1 Laboratório de Biologia Molecular de Artrópodes-LBMA-IB-RC-UNESP, Univ Estadual Paulista, Av. 24-A, n° 1515, Bela Vista, Rio Claro, SP CEP 13506-900, Brazil; bazonmurilo@gmail.com (M.L.B); aperezriverol@gmail.com (A.P.-R.); dmjacomini@gmail.com (D.L.J.-J.); 2 Centro de Estudos de Insetos Sociais-CEIS-IBRC-UNESP, Univ Estadual Paulista, Av. 24-A, n° 1515, Bela Vista, Rio Claro, SP CEP 13506-900, Brazil; jrbio04@rc.unesp.br (J.R.A.d.S.-P.); mspalma@rc.unesp.br (M.S.P.); 3 Laboratório de Imunologia Translacional-LIT, Departamento de Clínica Médica, Faculdade de Ciências Médicas, FCM, Universidade Estadual de Campinas-UNICAMP, Rua Tessália Vieira de Camargo n° 126, Cidade Universitária “Zeferino Vaz”, Campinas, SP CEP 13083-887, Brazil; luisgrf1982@gmail.com (L.G.R.F.); zollner@unicamp.br (R.d.L.Z.); 4 System Biology Department, Biomedical Research Division, Center for Genetic Engineering and Biotechnology, Ave. 31, e/ 158 and 190, P.O. Box 6162, Cubanacan, Playa, Havana 10600, Cuba; alexis.musacchio@cigb.edu.cu; 5 Centro de Estudos de Venenos e Animais Peçonhentos-CEVAP, Univ Estadual Paulista, Rua José Barbosa de Barros, 1780, Fazenda Experimental Lageado, Botucatu, SP CEP 18610-307, Brazil

**Keywords:** *Polybia paulista*, antigen 5, heterologous expression, allergy, diagnosis

## Abstract

*Polybia paulista* (Hymenoptera: Vespidae) is responsible for a high number of sting accidents and anaphylaxis events in Southeast Brazil, Argentina and Paraguay. The specific detection of allergy to the venom of this wasp is often hampered by the lack of recombinant allergens currently available for molecular diagnosis. Antigen 5 (~23 kDa) from *P. paulista* venom (Poly p 5) is a highly abundant and glycosylated allergenic protein that could be used for development of component-resolved diagnosis (CRD). Here, we describe the cloning and heterologous expression of the antigen 5 (rPoly p 5) from *P. paulista* venom using the eukaryotic system *Pichia pastoris*. The expression as a secreted protein yielded high levels of soluble rPoly p 5. The recombinant allergen was further purified to homogeneity (99%) using a two-step chromatographic procedure. Simultaneously, the native form of the allergen (nPoly p 5) was purified from the wasp venom by Ion exchange chromatography. The rPoly p 5 and nPoly p 5 were then submitted to a comparative analysis of IgE-mediated immunodetection using sera from patients previously diagnosed with sensitization to wasp venoms. Both rPoly p 5 and nPoly p 5 were recognized by specific IgE (sIgE) in the sera of the allergic individuals. The high levels of identity found between nPoly p 5 and rPoly p 5 by the alignment of its primary sequences as well as by 3-D models support the results obtained in the immunoblot. Overall, we showed that *P. pastoris* is a suitable system for production of soluble rPoly p 5 and that the recombinant allergen represents a potential candidate for molecular diagnosis of *P.paulista* venom allergy.

## 1. Introduction

Brazil hosts a wide diversity of clinically important Neotropical Hymenoptera including wasps, honeybee and ants. In the case of wasps, more than 320 species have been currently identified and several have been informed to coexist with human populations [1]. The venoms of these insects are mix of toxins including allergens that could induce local and systemic allergic reactions including life threatening anaphylaxis [2]. Early and accurate diagnosis of venom sensitization is critical for the success of immunotherapy which is the only causative treatment currently available [3]. In Brazil, the diagnosis of Hymenoptera venom allergy (HVA) has been based in the use of crude venom extracts and is often hampered by the incidence of cross-reactivity caused by the presence of cross-reactive carbohydrate determinants (CCDs) in allergen structures [4]. Molecular diagnosis based on the use of purified and extensively characterized recombinant allergens could help to overcome this pitfall resulting in proper identification of the primary sensitizing venom [5].

*P. paulista* (Hymenoptera: Vespidae), a Brazilian Neotropical wasp, has been informed to cause a high number of sting accidents along with dozens of fatal anaphylaxis cases annually [6,7]. The venom of this insect comprises three major allergens: phospholipase A1 (Poly p 1), hyaluronidase (Poly p 2) and antigen 5 (Poly p 5) [8]. These allergens were initially characterized using proteomic approaches [9,10,11]. Thus far, Poly p 1 [12] and Poly p 2 [13] have been cloned, expressed in *E. coli* and further evaluated in immunoblotting assays envisioning their use for development of CRD of *P. paulista* venom allergy. However, in both cases, the expression resulted in the production of the recombinant allergens as insoluble proteins, partially hindering the downstream characterization steps. The use of eukaryotic cells for heterologous expression could help to surpass this problem leading to the expression of a native-like soluble recombinant allergen [14,15,16].

The antigen 5 proteins belong to the CAP superfamily, which is composed of cysteine-rich secretory proteins (CRISP), antigen-5, and pathogenesis-related proteins (Pr 1). These proteins have been predicted to participate in diverse biological process such as reproduction, cancer, and allergic reactions [17]. Antigen 5 proteins have been successfully used in molecular diagnosis from differentiation of true double sensitization to wasp and honeybee venoms from cross-reactivity [18]. As CCD-lacking proteins that have been identified exclusively in wasp and ant venoms, they prevent the incidence of cross-reactivity with HBV-elicited sIgE [18,19]; And in the wasp venoms, the antigen 5 proteins have shown high sensitivity rates, up to 96% combined with phospholipase [20,21] which is an important issue in molecular diagnosis. 

Similar to other wasps, antigen 5 from *P. paulista* (~23 kDa) is a highly abundant, glycosylated allergen with unknown biological function [11,22]. The nPoly p 5 is a predominant venom component [8,11,23] that exist as six multiple forms in the crude venom [8]. Alignment of the native allergen primary sequence showed different levels of homology with other annotated vespid venoms ranging from 59.3–93.7% of identity [11]. These levels of homology in primary sequence represent the basis for the cross-reactivity observed among antigen 5 from different wasps [18]. A recently published analysis with the antigen 5 [23] showed the presence of nine linear B-cell epitopes immunoreactive to human IgG and at least one immunoreactive to human IgE that could be responsible for eliciting sIgE antibodies in allergic patients. 

To date, venoms antigen 5 have been produced in *E. coli* [24,25], yeast [16] and insect cells [18]. While the use of bacterial system resulted in the production as insoluble protein, the expression in eukaryotic systems has showed to be highly suitable from production of soluble secreted forms of the allergen with a native-like fold and, thus, allergenic profile. Here, we describe the soluble expression of rPoly p 5 in *Pichia pastoris* X-33 cells. High yields of the secreted recombinant protein were obtained in the fermentation broth with induction at 28 °C, (1%) methanol after 120 h. A comparative analysis IgE-mediated immunodetection of the purified rPoly p 5 and nPoly p 5 was conducted using sera from allergic patients. The results described here strongly suggest that rPoly p 5 is a potential candidate for development of molecular diagnosis of *P. paulista* venom allergy. 

## 2. Results

### 2.1. Recombinant Poly p 5 Coding Sequence

**Figure 1:**
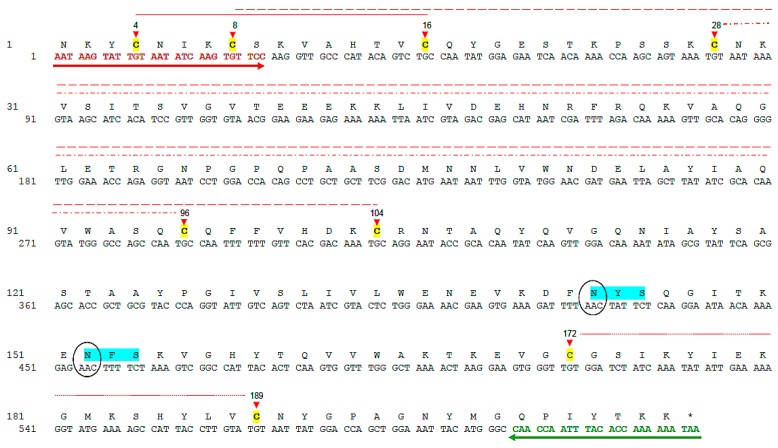
Nucleotide and predicted amino acid sequences of rPoly p 5 (Gen Bank: ANW82807.1) Forward (red arrow) and reverse (green arrow) primers used for gene specific amplification are indicated. Cysteine residues potentially involved in disulfide bridges (discontinuous red lines) are highlighted in yellow and the position marked with red triangles. The consensus sequons for N-glycosylation are in light blue with the asparagine residue enclosed in black ovals. (For interpretation of the references to color in this figure legend, the reader is referred to the web version of this article.)

After sequencing of five *poly p 5*_pCR^®^8/GW/TOPO^®^ positive clones a unique consensus sequence (612 bp) was obtained and annotated (GenBank: KU558986.1). A multiple alignment of the obtained sequence using the Blastn tool [26] showed different levels of identity with native antigen 5 from other clinically relevant Hymenoptera including *P. scutellaris* (nPoly s 5) (99%), *P. annularis* (nPol a 5) (85%), *P. dominulus* (nPol d 5) (82%), *V. vulgaris* (nVes v 5) (74%), *D. maculate* (nDol m 5) (74%) and *S. invicta* (nSol i 3) (68%). The putative primary sequence of rPoly p 5 (GenBank: ANW82807.1) contains 206 amino acids with eight cysteine residues, all potentially involved in disulfide bridges formation (Cys4-Cys16, Cys8-Cys104, Cys28-Cys96, Cys172-Cys189) (Figure 1) in accordance with Dos Santos-Pinto et al., [11]. The analyses of the predicted primary sequence using the Compute pI/MW tool [27] showed a theorical pI value of 8.9 and a molecular weight of 23.08 kDa. Meanwhile, in silico prediction for N-glycosylation sites using the NetNGlyc 1.0 Server [28] showed two consensus sequons (Asn-Xaa-Ser/Thr) at position N143 and N152. However, this analysis also showed that none of these residues are predicted to be glycosylated. 

**Figure 2:**
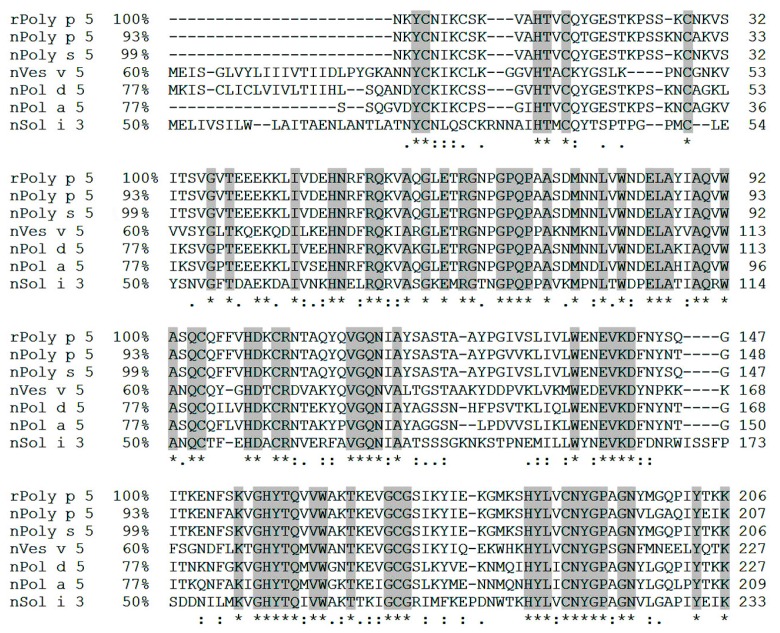
Multiple alignment and identity values among the primary sequence of the predicted rPoly p 5 protein (GenBank: ANW82807.1) with the primary sequences of nPoly p 5 (*Polybia paulista*), nPoly s 5 (*Polybia scutellaris rioplatensis*), nVes v 5 (*Vespula vulgaris*), nPol d 5 (*Polistes dominula*), nPol a 5 (*Polistes annularis*), and nSol i 3 (*Solenopsis invicta*). The most important regions of identity are marked in dark gray, highlighting the highly conserved residues in this type of alignment.

In contrast with the results informed for Poly p 1 [12], the predicted primary sequence of rPoly p 5 showed moderate to high levels of identity with antigen 5 from other Hymenoptera venoms, ranging from 50% in relation to Sol i 3 and 99% in relation to Pol s 5 (Figure 2). These levels of identity have been identified as the main cause of cross-reactivity among antigen 5 sequences [18] and could influence the results of diagnosis using rPoly p 5 as a solely marker for differentiation of wasp and ant venom sensitizations.

### 2.2. Heterologous Expression and Purification of rPoly p 5

**Figure 3:**
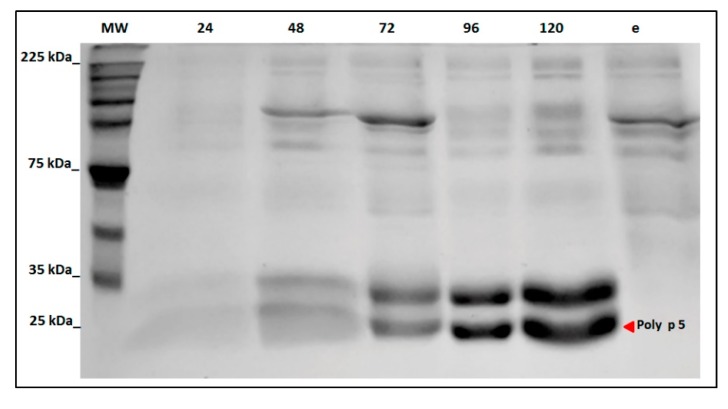
SDS-PAGE (12%) of the supernatant from *P. pastoris* X-33 culture during rPoly p 5 expression. Abbreviations: 24–120, hours after induction (1% methanol); e, negative control (*P. pastoris* X-33 transformed with an empty pPICZαA vector).

The supernatant of the *P. pastoris* X-33 culture was analyzed for detection of soluble rPoly p 5. A band of ~23 kDa which corresponds to the predicted molecular weight was detected in SDS-PAGE as soon as 24 h after induction (1% methanol) (Figure 3). The higher levels of expression were obtained at 120 h. The expression of rPoly p 5 under optimal conditions (28 °C, 1% methanol with shaking at 250 rpm) yielded a maximum of 358 mg per liter of crude supernatant. Interestingly, a second band of ~33 kDa with equimolar concentration was also detected during the expression. 

### 2.3. Recombinant Allergen Purification

**Figure 4:**
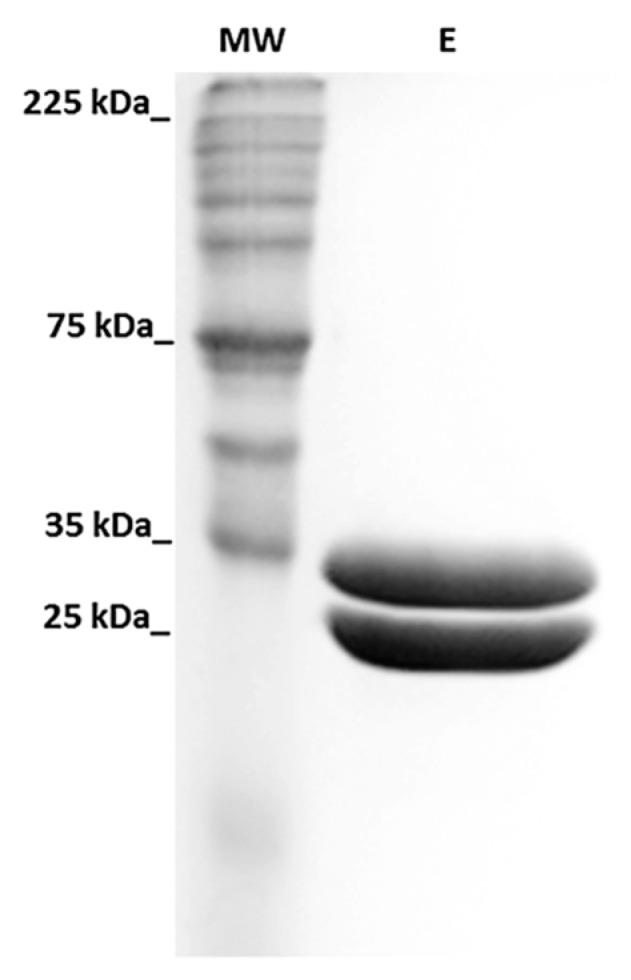
SDS-PAGE (12%) analysis of fractions eluted at 75 mM during rPoly p 5 purification by IMAC. E, Elution.

After production of rPoly p 5 under the optimal expression conditions the supernatant was filtered and applied to a HisTrap HP™ (GE Healthcare, Uppsala, Sweden) commercial prepacked column for affinity chromatography using the N-terminal 6XHis tag provided by the pPICZαA. The analysis of the collected fractions showed that rPoly p 5 eluted at a concentration of 75 mM imidazole. Two bands were obtained after elution, one with the expected molecular weight (~23 kDa) and the other with ~33 kDa (Figure 4) which represents a modified variant of the recombinant allergen. The appearance of this variant could be due either to a hyper-mannosylation of the rPoly p 5 [16] or misprocessing of the α mating factor (α-MF) secretion signal encoded in the pPICZαA from recombinant protein secretion. The molecular basis for the origin of this second band will be discussed latter in this work. 

**Figure 5:**
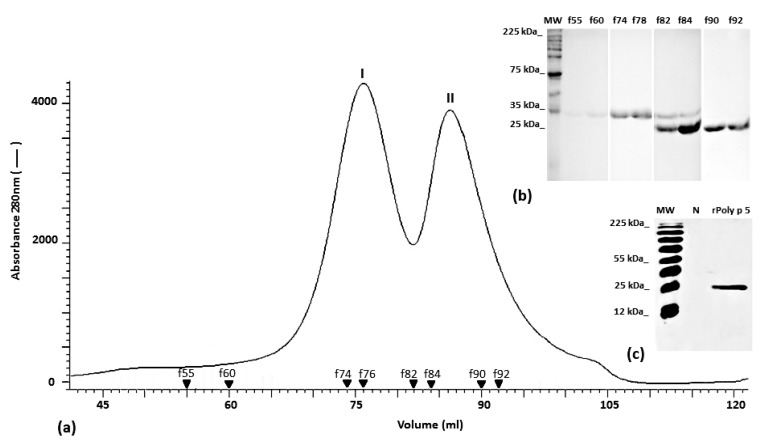
Sephadex G-100 size-exclusion chromatography profile of the sample eluted from Ni^2+^ Sepharose affinity chromatography (**a**). The chromatography was conducted with 5 mM ammonium acetate pH 6.8 buffer and 1 mL fractions were collected at a flow rate of 1 mL/min. Protein elution was monitored at 280 nm (___). The band profile in (12%) SDS-PAGE of the most representative fraction (numbered and marked with black triangles) is shown (**b**) along with the immunodetection of the purified rPoly p 5 using anti-His antibody (**c**).

To purify the rPoly p 5 band corresponding to the expected molecular weight, the fractions obtained after the affinity chromatography were pooled and submitted to size exclusion on a Sephadex G-100 coupled to an ÄKTA-FPLC system. All fractions from the two peaks obtained after the chromatographic separation (Figure 5a) were analyzed by (12%) SDS-PAGE. In Figure 5b, the protein band profiles of the more representative fractions are show. A single band with the expected molecular weight (~23 kDa) for rPoly p5 was obtained in fractions f90 to f92. The identity of the purified protein was also assessed by Western blotting using an anti His-antibody (Figure 5c). 

**Figure 6:**
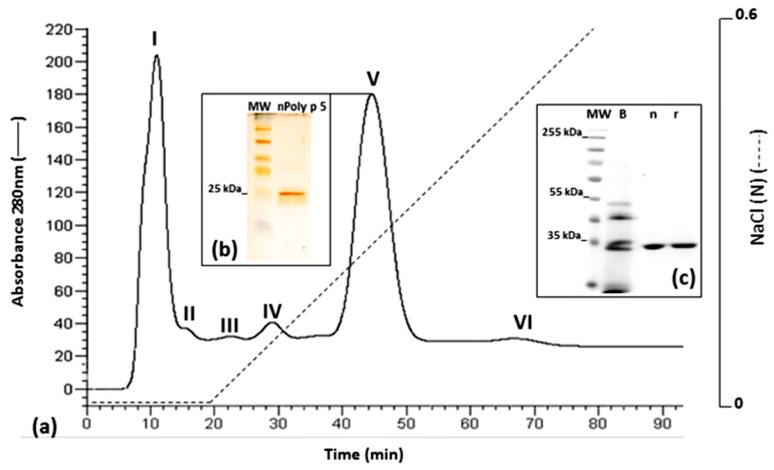
Purification of native Poly p 5 from *P. paulista* venom. Cation-exchange chromatography profile obtained after crude venom extract fractionation, using a Hiprep FF CM column (16 × 10 mm, 20 mL; GE Healthcare) coupled to an AKTA-FPLC system, similar to previously obtained [12,26]. The elution was performed under a linear gradient from 0 to 1 M NaCl (_ _ _). The protein was monitored by measuring the absorbance at 280 nm, represented by a continuous line (___) (**a**). Two-milliliter fractions were collected for further detection of nPoly p 5 (~25 kDa) by 12% SDS-PAGE of all fractions eluted, and the gels stained with silver stain. A representation of the SDS-PAGE profile showing a unique band of ~25 kDa obtained from the main fractions (20–25) of the peak V (**b**). The crude venom (B), the purified native (n) and recombinant (r) Poly p 5 were finally analyzed in a (12%) SDS-PAGE (**c**).

A single-step cation exchange chromatographic protocol was used to purify nPoly p 5. The profile obtained after venom separation (23 mg) was similar to those previously informed [12,29] (Figure 6a). After the separation, all fractions were analyzed by (12%) SDS-PAGE. A single band with the expected molecular weight (~25 kDa) for nPoly p 5 was detected in peak V (fractions 20–25), which is one of the two major peaks detected in the chromatogram. This result agrees with previous studies, in which antigen 5 from *V. vulgaris* was reported as a highly abundant component, accounting for 5–10% of the venom dry weight [4]. Figure 6b shows the band profile in a (12%) SDS-PAGE of a representative fraction (22) containing the purified nPoly p 5. 

### 2.4. IgE-Mediated Immunodetection 

**Figure 7:**
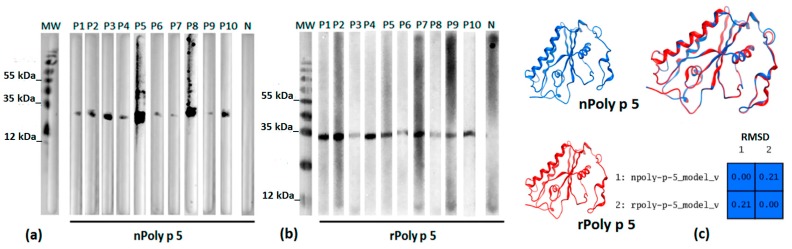
IgE-mediated immunodetection of native (**a**) and recombinant (**b**) Poly p 5. Serum samples: P1-P10 = individual sera from patients previously diagnosed with specific IgE to P. paulista venom; N = pool of five serum samples from nonsensitized healthy volunteers, as negative control. The molecular weight marker (kDa) is indicated. The results of (**a**,**b**) are supported by the in silico analyses of the 3-D models of nPoly p 5 and rPoly p 5 showing the high structural homology between them (**c**) which is predicted by the presence of commons linear and conformational epitopes. The root-mean-square deviation (RMDS) of atomic positions of the 3-D models from nPoly p 5 and rPoly p 5 compared to the template 3-D model (Ves v 5, PDB 1QNX) is showed in the blue box.

The native and the recombinant Poly p 5 were analyzed by immunoblotting for IgE-mediated immunodetection. The sera from ten patients previously diagnosed with allergy specific to wasp venoms, by the ImmunoCAP system (Phadia, Sweden), recognized both purified forms—rPoly p 5 (peaK II, Figure 5) and nPoly p 5 (peak V, Figure 6). A single band of molecular mass ~23 kDa was detected in both cases (Figure 7) while no detection occurred with sera of non-sensitized patients. This result confirms that fractions obtained from peak V really correspond to native allergen. In addition, the results obtained in the immunoblot agree with those obtained in Figure 2 for the alignment of primary sequences of nPoly p 5 and rPoly p 5 and also for the 3-D models (Figure 7c) of both forms, in which high levels of identity were detected. The IgE-mediated recognition demonstrated here is predicted by the presence of commons linear and conformational epitopes in the native and the recombinant forms of the allergen. Although a greater number of sera and other types of analysis such as basophil activation test are needed to extent the reliability of these results, this primary immunological evaluation pointed to high levels of sensitivity (100% recognition in the allergic sera tested) associated to the use of rPoly p 5, reinforcing the idea that this recombinant allergen could be used to improvement of molecular diagnosis of venom allergy. 

## 3. Discussion

Specific diagnosis of HVA is a critical step for starting venom immunotherapy. Miss identification of culprit insect could result in the inclusion of irrelevant venoms in the treatment and the de novo sensitization of the patients. Despite the wide diversity of Hymenoptera identified in Neotropical regions, the diagnosis of allergy is based on skin tests and in vitro sIgE detection using venom extracts, which is related to significant levels of cross-reactivity. The production of recombinant allergens from venom of endemic species is a main goal in order to develop molecular diagnosis and increase the feasibility of the procedures currently used for identification of the primary sensitizing venom. In the case of *P. paulista*, a systematic approach that includes proteome analysis for toxins identification and their molecular characterization has allowed the subsequent cloning and recombinant production of the major allergens of the insect venom [1].

Here, we reported the soluble expression and the IgE-mediated recognition of the antigen 5 from *P. paulista* venom. In some Hymenoptera venoms antigen 5 is related as primary marker of venom allergy due to the lacking of CCDs, of homologues in HBV and their overrepresentation in wasp and ant venoms [18]. Most of the system currently used for diagnosis of wasp allergy and differentiation of double sensitization from cross-reactivity are based in the use of this allergen. Recombinant production of antigen 5 often resulted in high levels of protein yields, regardless the cells system used for expression. Although *E. coli* remains a highly popular system for heterologous expression, its use is hampered by protein aggregation and miss folding, which finally affect the downstream use of the product [30]. Considering the results previously obtained by our group for the expression of *P. paulista* major allergens, Poly p 1 [12] and Poly p 2 [13] in *E. coli* which resulted in insoluble products, we aimed the expression of Poly p 5 in the eukaryotic system *P. pastoris*. The use of this yeast allowed the production of secreted and native-like soluble allergens [31]. Furthermore, *P. pastoris* is an easy-to scale system that facilitates the downstream production of large amounts of the desire protein. 

As expected, high levels of expression of the rPoly p 5 (~23 kDa) were obtained in the fermentation broth after induction with (1%) methanol. As the *P. pastoris* X-33 strain and expression conditions tested initially resulted in protein yields of 358 mg per liter which are significantly higher to previous report in Pol s 5 (25 mg per liter or 14 folds) [16], no further analysis for expression optimization were conducted. The rPoly p 5 could be clearly detected in SDS-PAGE 48 h after the initial induction. The levels of the secreted recombinant allergen observed in the supernatant increase until 120 h post-induction (Figure 3). 

A second band (~35 kDa) was also detected in the collected fractions. This result is similar to previously reports in Poly s 5 [16] in which a second band was detected during expression in *P. pastoris*. Further analysis conducted in that study showed that this band corresponds to a glycosylated form in residue N^144^ of the recombinant protein. Considering the levels of the identity between Pol p 5 and Pol s 5 primary sequences and the conservation of the Pol s 5-glycosylated site in Poly p 5 (N^143^), this second band should correspond to a more glycosylated form of Poly p 5. It has been extensively reported and discussed that one of the major drawbacks of the recombinant expression in yeast is the unwanted hyper-mannosylation of the target protein [32]. As nPoly p 5 has a molecular weight of 25 kDa, we aimed to obtain rPoly p 5 with a similar structural and immunological profile to its native counterpart. Thus, we used a sequential two-step chromatographic procedure to purify the band of 25 kDa of the recombinant allergen before conducting the IgE-mediated immunoreactivity analyses. 

The appearance of the non-specific ~35 kDa band could be also related to a failure in processing the secretion signal (α-MF) by *P. pastoris* X-33 cells. It has been reported that overexpression of recombinant proteins in yeast, due to the high efficiency of the P_AOX_ promoter, often results in misprocessing of the secretion signal [33]. In fact, the expression of a hybrid allergen containing parts of *V. vulgaris* and *P. annularis* antigen 5s resulted in a differential processing of the N-terminal signal [34]. Interestingly, we found that the difference between the molecular weights of the two bands obtained after IMAC purification (Figure 4) matches with the molecular weight of the (α-MF) secretion signal (~10 kDa). However, further analyses of the N-terminal from the rPoly p 5 variants obtained should be conducted in order to identify the molecular basis of the second band detected in this study. 

After the sequential chromatographic steps the rPoly p 5 variant matching with the molecular weight of the nPoly p 5 was purified to homogeneity. The recombinant allergen was extensively immunodetected by allergic patients’ sera. However, the chromatographic separation of the proper rPoly p 5 from the other band resulted in a significant decrease (158 mg per liter, 44%) of recombinant allergen levels. Rational-designed alternatives are needed in order to avoid either N-mannosylation or misprocessing of the N-terminal of the expressed rPoly p 5 to finally increase the protein yields. At least for Poly s 5 [16], the authors found that a mutation in N^144^S residue completely prevented the heterologous protein glycosylation. Interestingly, the mutated N^144^S_Poly s 5 variant was immunologically active and showed an nPoly p 5-like profile on IgE-binding inhibition assays. On the other hand, expression at low temperatures, with low aeration or the use of moderately expressing promoters that prevent protein overproduction could be used to avoid failure in signal processing [35]. 

Immunoblotting analysis showed that secreted and further purified rPoly p 5 is extensively recognized by sera of patients previously diagnosed with sIgE to wasp venoms. The immunodetection indicates that the recombinant allergen retains the linear, native-like IgE epitopes and consequently represents a potential candidate for development of molecular-defined diagnosis of allergy. In the case of discontinuous and conformational epitopes, further analyses using ELISA and/or BATs should be conducted in order to evaluate their conservation in the heterologous protein. However, considering the results reported for the close-related Poly s 5 [16], an nPoly p 5-like profile of conformational epitopes is also expected. The in silico analyses using the predicted 3-D models of the native and the recombinant allergens showed high levels of homology, suggesting that the properly folded rPoly p 5, produced in *P. pastoris* contains the sIgE-clinically relevant epitopes. 

Interestingly, even when the use of immunoblotting analysis could hamper the detection of conformational epitopes, 100% of the sera reacted with rPoly p 5. The use of recombinant antigen 5 (rVes v 5) in allergy diagnosis is associated to high levels (90%) of sensitivity [21]. Meanwhile, spiking venom extracts with Ves v 5 has resulted in up to 96.8% of allergic patients identification [36]. The higher levels of sensitivity demonstrated here are related to the limited number of sera analyzed and the differences in the semi-quantitative procedure used. Further studies along with extensive immunological assays are require in order extend this data. However, the preliminary analysis conducted here along with the results informed for Poly p 1 [12] suggests that a combination of these two major allergens could result in levels of sensitivity similar or even higher to those obtained with conventional venom extract. 

## 4. Concluding Remarks

Overall, herein we informed the high levels of expression of a secreted rPoly p 5 using the methylotrophic yeast *P. pastoris* X-33 cells. This system represents a good alternative for heterologous expression of this allergen as a soluble allergen and could be used in large scale fermentation for protein production. The rPoly p 5 was purified to homogeneity by immobilized metal ion affinity chromatography in combination with size exclusion chromatography. The immunoreactivity of purified rPoly p 5 was assessed with a panel of sera from patients sensitized to wasp venoms. The analysis revealed a high degree of sensitivity associated to the use of this recombinant allergen. With the production of immunologically active rPoly p 5, the complete set of major allergens from *P. paulista* venom is now available as recombinant proteins. Further evaluations of these allergens are currently being conducted envisioning the development of panels of Poly p 1, Poly p 2 and Poly p 5 that could result in the development of molecular diagnosis of *P. paulista* venom allergy. 

## 5. Materials and Methods

### 5.1. Sera from Allergic Patients

Sera from 10 patients, regardless sex or age, with a history of allergic reactions to Hymenoptera and previously diagnosed with sIgE to wasp venoms were obtained from the Ambulatório de Anafilaxia of the Hospital das Clínicas, Faculdade de Ciências Médicas, Universidade Estadual de Campinas-UNICAMP. A pool of five sera from non-sensitized healthy volunteers was used as negative controls. The study was approved by the Ethics Committee of FCM-UNICAMP under No. 187/2006 (23 July 2006 and updated on 23 September 2008). Informed consent was obtained in written form from all participants of the study, and participation was voluntary. 

### 5.2. Polybia paulista Venom Collection

The glands from *P. paulista* were obtained as previously described by Perez Riverol et al., 2016 [12]. Briefly, *P. paulista* nests were captured around or within the campus of Universidade Estadual Paulista “Júlio de Mesquita Filho” (UNESP), Rio Claro, SP, Brazil; licensed of SisBio (Protocol number: 58500). After taxonomical identification, the collected wasps were immediately anesthetized at low temperature (−80 °C), and their venom glands (gl) were extracted using sterile tweezers. Two hundred glands were used for RNA extraction while 3000 glands were used for nPoly p 5 purification. 

### 5.3. Poly p 5 cDNA Cloning and Sequencing

For RNA extraction the venom glands were gently washed with mili-Q sterile water, suspended in 200 μL of Trizol^®^ (Life Technologies, Carlsbad, CA, USA), macerated and then stored at −80 °C for a week. The first-strand cDNA synthesis was performed using 1 μg of total RNA extracted, the oligo dT-primer 5′-GGCCACGCGTCGACTAC(T)_17_-3′ adapter (Gibco-Life Technologies, Carlsbad, CA, USA), and the ImProm-II Reverse Transcription kit (Promega, Madison, WI, USA), following the manufacturer’s instructions. The *npoly p 5* cDNA amplification was performed using the PCR Master Mix (Qiagen, Hilden, Germany) and the primers poly p 5 *forward* (5’-AATAAGTATTGTAATATCAAGTGTTCCAAG-3’) and AMP-*reverse* (5’-GGCCACGCGTCGACTAC-3’) (LifeTechnologies, São Paulo, Brazil). The following conditions were settled for reaction: 5 min at 95 °C; 35 × 95 °C, for 1 min, 55 °C for 3 min, and 72 °C for 3 min; and a final extension of 72 °C for 5 min. After analyzed in 1% agarose gel in 1XTAE (40 mM Tris acetate, 1 mM EDTA, pH 8.3) buffer, the PCR products were purified and cloned on pCR^®^8/GW/TOPO^®^ vector (kit pCR^®^8/GW/TOPO^®^—Invitrogen, Waltham, MA, USA) according to the manufacturer’s instructions. The DNA was confirmed by sequencing using the primers GW1 forward (5′-GTTGCAACAAATTGATGAGCAATGC-3′) and GW2 reverse (5′-GTTGCAACAAATTGATGAGCAATTA-3′) in an Applied Biosystems 3730 sequencer at the Center for Study of Social Insects (CEIS, UNESP, Rio Claro, SP, Brazil). The resulting sequences were analyzed and edited using the DNASTAR^®^ Lasergene Sequence Analysis software [34].

### 5.4. Poly p 5 Recombinant Expression 

The nucleotide sequence codifying for *npoly p 5* cDNA (GenBank: ANW82807.1) was chemically synthetized after codon optimization [37] for expression in *P. pastoris*. The sequence was then cloned in pPICZαA vector and the resulted pPICZαA_*poly p 5* construct was used to transform *P. pastoris* X-33 cells. All the procedures described were conducted by GenScript (Piscataway, NJ, USA). The *P. pastoris* X-33_*poly p 5* transformants were then selected using YPD-agar plates (1% yeast extract, 2% peptone, 2% dextrose, and 2% agar) supplemented with 100 μg/mL of Zeocine (Invitrogen, Waltham, MA, USA). 

For the Poly p 5 recombinant expression, a single colony from transformants was used to inoculate in 25 mL of BMGY (buffered minimal glycerol medium, recipe from Invitrogen). The pre-inoculum was incubated overnight at 28 °C and 250 rpm. The OD_600_ of the culture was measured and a suitable volume was centrifuged at 3000× *g*, 4 °C for 10 min to suspended in 100 mL of BMMY (buffered minimal methanol medium) at a final OD_600_ = 1.0. The BMMY medium was transferred to a 1 L baffled shake flask and incubated for 120 h, at 28 °C with daily addition of (1%) methanol. Aliquots of 2 mL were withdrawn each day and further evaluated to determine the optimal expression time. At the end of the induction, the cultures were centrifuged at 4 °C, 10.000× *g* for 10 min and the cleared supernatant was collected for analysis in (12%) SDS-PAGE

### 5.5. Purification of the Recombinant and Native Poly p 5 

After concentration, the rPoly p 5 was purified by immobilized metal ion affinity chromatography, with a HisTrap HP™ (Ni^2+^ Sepharose™ High Performance; GE Healthcare, Uppsala, Sweden), according the manufacturer’s instructions. Briefly, the supernatants were sequentially filtered (0.45 and 0.22 μm membranes), concentrated and suspended in sodium phosphate buffer (equilibration buffer—20 mM NaH_2_PO_4_, 20 mM Na_2_HPO_4_, 20 mM Imidazol; pH 7.4) using and Amicon^®^ Ultra-15 centrifugation filters 10 K (Merck Millipore, Billerica, MA, USA). The proteins were applied to the system coupled with a peristaltic pump (GE Healthcare, Uppsala, Sweden). The column was then washed sequentially with 10 mL of each: (a) equilibration buffer without imidazole; (b) with 25 mM; and (c) with 50 mM imidazole. Elution of the 6XHis-tagged rPoly p 5 was performed with 10× the column volume with the elution buffer (the same as the equilibration buffer but containing 75 mM imidazole). Five fractions of 2 mL were collected at a flow rate of 1 mL/min. All the collected fractions were further analyzed by (12%) SDS-PAGE.

For separation of the rPoly p 5, the fractions obtained from the affinity chromatography were pooled, liophylized, resuspended in 5 mM ammonium acetate pH 6.8 and submitted to a size-exclusion chromatography in a Sephadex G-100 resin (90 × 2.5 cm) coupled to an ÄKTA-FPLC system (GE Healthcare, Uppsala, Sweden). The column was previously equilibrated with the same buffer. Fractions of 1 mL were collected at a flow rate of 1 mL/min. The elution was monitored by measuring the absorbance at 280 nm and the fractions were analyzed for the presence of purified rPoly p 5. Finally, the nPoly p 5 was purified using crude venom (23 mg) from 3000 glands of *P. paulista* following the one-step cation-exchange chromatographic procedure as previously described for n Poly p 2 and nPoly p 1 [12,26]. 

### 5.6. SDS-PAGE and Protein Quantification 

SDS-polyacrylamide gel electrophoresis (SDS-PAGE) 12% was performed according to Laemmli (1970) [38], using a Mini-Protean^®^ Tetra Cell System (BioRad, Hercules, CA, USA). After running, the gels were stained either with Coomassie Brilliant Blue R-250 (CBB) or silver stain (Thermo Fisher Scientific, Waltham, MA, USA). Protein in crude venom and purified native or recombinant allergens were quantified by the modified Bradford method [39] using bovine serum albumin (Sigma, St. Louis, MO, USA) as a standard. 

### 5.7. Anti-His Immunodetection and Immunoblotting Analyses 

The purified rPoly p 5 was detected with an Anti-His Antibody Selector Kit” (Qiagen, Germantown, MD, USA) according to manufacturer instructions using a rat anti-mouse IgG (subclass IgG1) conjugate to horseradish peroxidase (1:500). The bands were visualized in Image Quant 400 (GE Healthcare, Uppsala, Sweden) using the chemiluminescent substrate Luminata™ ForteWestern HRP substrate (Millipore, Billerica, MA, USA).

The in vitro recognition of rPoly p 5 and nPoly p 5 by sIgE antibodies in sera from allergic patients (10) were conducted using the protocol previously described by Perez-Riverol et al., 2016 [12]. Briefly, 30 µg of the allergens were submitted to SDS-PAGE and electrotransferred to a 0.22 mm nitrocellulose membrane using a semi-dry system (Trans-Blot^®^ SD Semi-Dry Electrophoretic Transfer Cell, Bio-Rad, Hercules, CA, USA). After blocking with 20 mM Tris-HCl, 150 mM NaCl, pH 7.4, with 0.5% Tween-20 (Sigma-Aldrich, St. Louis, MO, USA) (TBS-T wash solution) and 3% non-fat dried milk (block solution) for 1 h at room temperature under slow agitation on a Rocker II™ Platform mixer (Boekel Scientific, Feasterville-Trevose, PA, USA), the membranes were washed (3 times with TBS-T wash solution). The membranes were then incubated overnight with 400 μL of each patient’s sera (1:50) using a mini PROTEAN^®^ II multi-screen apparatus (Bio-Rad, Hercules, CA, USA). Immunodetection was performed using anti-human IgE (ε-chain specific) peroxidase conjugate antibody (Sigma-Aldrich, St. Louis, MO, USA) (1:5000). The bands were visualized as described in Section 5.6.

### 5.8. Molecular Modelling 

The 3-D models of rPoly p 5 and nPoly p 5 were generated by MODELLER [40] using the crystal structure of the venom antigen 5 from *Vespula vulgaris* (PDB 1QNX) as general template. The model was subjected to energy minimization using YASARA software [41] and then validated using MolProbity [42]. The ribbon diagram was prepared with PyMOL [43] using the homology models as input.

## Abbreviations

Poly p 5	Antigen 5 from *Polybia paulista* venom
Poly p 1	phospholipase A1 from *Polybia paulista* venom
CCDs	Cross-reactive carbohydrate determinants
HVA	Hymenoptera venom allergy
HBV	Honeybee venom
CRD	Component resolved diagnosis
